# Genomic comparison of two independent seagrass lineages reveals habitat-driven convergent evolution

**DOI:** 10.1093/jxb/ery147

**Published:** 2018-04-18

**Authors:** HueyTyng Lee, Agnieszka A Golicz, Philipp E Bayer, Anita A Severn-Ellis, Chon-Kit Kenneth Chan, Jacqueline Batley, Gary A Kendrick, David Edwards

**Affiliations:** 1 School of Agriculture and Food Sciences, University of Queensland, Brisbane, QLD, Australia; 2 School of Biological Sciences, University of Western Australia, WA, Australia; 3 Plant Molecular Biology and Biotechnology Laboratory, Faculty of Veterinary and Agricultural Sciences, University of Melbourne, Parkville, Melbourne, VIC, Australia

**Keywords:** Gene loss, *Halophila ovalis*, marine adaptation, NDH complex, osmoregulation, seagrass, *Zostera muelleri*

## Abstract

Seagrasses are marine angiosperms that live fully submerged in the sea. They evolved from land plant ancestors, with multiple species representing at least three independent return-to-the-sea events. This raises the question of whether these marine angiosperms followed the same adaptation pathway to allow them to live and reproduce under the hostile marine conditions. To compare the basis of marine adaptation between seagrass lineages, we generated genomic data for *Halophila ovalis* and compared this with recently published genomes for two members of Zosteraceae, as well as genomes of five non-marine plant species (Arabidopsis, *Oryza sativa*, *Phoenix dactylifera*, *Musa acuminata*, and *Spirodela polyrhiza*). *Halophila* and Zosteraceae represent two independent seagrass lineages separated by around 30 million years. Genes that were lost or conserved in both lineages were identified. All three species lost genes associated with ethylene and terpenoid biosynthesis, and retained genes related to salinity adaptation, such as those for osmoregulation. In contrast, the loss of the NADH dehydrogenase-like complex is unique to *H. ovalis*. Through comparison of two independent return-to-the-sea events, this study further describes marine adaptation characteristics common to seagrass families, identifies species-specific gene loss, and provides molecular evidence for convergent evolution in seagrass lineages.

## Teaser

In adapting to a marine environment, two independent seagrass lineages lost genes associated with ethylene and terpenoid biosynthesis and retained genes related to salinity adaptation, suggesting habitat-driven convergent evolution.

## Introduction

Seagrasses are a polyphyletic group of flowering plants that live fully submerged in the marine environment and form monospecific meadows resembling terrestrial grasses. The morphology of seagrasses varies among species, though common features include long, strap-shaped leaves and simple flowers. Seagrasses belong to a basal lineage that diverged around 140 million years ago (Mya), before the divergence of the Poaceae within the monocotyledon clade. Although similar in form, seagrass species represent at least three independent return-to-the-sea events (Les *et al.*, 1997).

The convergent evolution of seagrasses is characterized by common physiological and morphological features that possibly represent a collection of marine adaptation traits. For example, seagrass leaves lack stomata, and gas exchange occurs through permeable cuticles, while seagrass roots and rhizomes have aerenchyma to enhance gas transport. Seagrasses have also adapted to variable quality and low levels of light, which attenuates quickly in seawater (Larkum *et al.*, 2006; Strydom *et al.*, 2017) and have effective osmoregulation to survive in the saline aqueous environment (Koch *et al.*, 2007; Touchette, 2007). Seagrasses are adapted to aquatic reproduction, where the transport and capture of pollen grains is carried out on or below the water surface.

Current seagrass taxonomy contains around 72 species forming three families, Zosteraceae, Hydrocharitaceae and Cymodoceaceae complex (Les *et al.*, 1997; Short *et al.*, 2011; Nguyen *et al.*, 2015). Recent genome-wide comparative studies of two species in the Zosteraceae provided the first insight into genomic adaptation to the marine environment (Golicz *et al.*, 2015; Lee *et al.*, 2016; Olsen *et al.*, 2016). Genes associated with the synthesis and signalling of volatile substances, including ethylene, methyljasmonate, and terpenoids, were lost in both *Zostera muelleri* and *Z. marina*. Genes associated with morphological adaptation, including those for stomatal cell differentiation, flower development and pollen formation, were also absent or greatly reduced in number. An increase in gene families associated with low light harvesting and cell wall modification was observed and postulated to contribute to survival in the light-attenuated and high salinity environment.

These gene losses, gene modifications, and gene family expansions in the two *Zostera* species may not reflect the independent adaptation of other seagrass lineages to the marine environment, and analysis of a second lineage is required to answer the question whether they share a common adaptation pathway to the ocean. *Halophila ovalis* is a seagrass species in the family Hydrocharitaceae, and is an ideal model for comparison with the *Zostera* species. The seagrass subclade in Hydrocharitaceae is embedded within branches of largely diverse aquatic angiosperms, including freshwater species (Larkum *et al.*, 2006), indicating the independent rise of marine adaption phenotypes.

As the likelihood of convergent evolution is predicted to decrease with phylogenetic distance (Ord and Summers, 2015), the divergence time difference between the seagrass subclade in Hydrocharitaceae (55 Mya; Chen *et al.*, 2012) and Zosteraceae (25 Mya; Coyer *et al.*, 2013) highlights the importance of this study. Moreover, since examples of parallel evolution, where similar phenotypes are generated from a similar genetic process of independent convergent evolution (Ord and Summers, 2015), are not abundant in plants (examples include carnivorous species (Fukushima *et al.*, 2017), recurrence of C4 photosynthesis (reviewed in Washburn *et al.*, 2016) and convergent mutations in loci during domestication (Paterson *et al.*, 1995)), and that habitat is the most common factor associated with reported examples of repeated evolution (Ord and Summers, 2015), independent seagrass lineages are excellent subjects for study.

In this work, a genomic comparison between seagrasses of Hydrocharitaceae and Zosteraceae was explored to determine whether the gene loss previously identified in *Z. muelleri* and *Z. marina* is also observed in *H. ovalis*. We also attempt to identify any seagrass-specific genes that are present in one or both lineages. *Halophila ovalis* genome sequencing data were compared with the annotated genomes of *Z. marina* and *Z. muelleri*, together with representative land plants. Our study demonstrates that lost genes associated with the synthesis and signalling of volatile substances, as well as stomatal development, are shared by both seagrass lineages. Genes that are uniquely conserved across the two lineages are enriched in pathways related to cell osmoregulation, and provide molecular evidence for independent marine colonization. Results also revealed the loss of the NADH dehydrogenase-like (NDH) protein complex in *H. ovalis*, a characteristic that is not shared by the other two seagrass species. This study provides a more complete description of marine adaptation, and suggest a parallel convergent evolution of two independent return-to-the-sea events in seagrasses separated by 30 million years.

## Materials and methods

### Genome sequencing of *H. ovalis*

One *H. ovalis* plant sample was collected at Swan River, Claremont, Perth, Western Australia (coordinates: 32° 0′ 3.98″ S, 115° 45′ 18.31″ E).

The growth tips of the seagrass thalli were carefully removed, rinsed in sterile water, and inspected for visible external contamination. Seven hundred milligrams of tissue was placed in 5 ml tubes, flash frozen in liquid nitrogen, and bead-pulverized using a 2010 Geno/Grinder (SPEX SamplePrep, USA). The Qiagen DNeasy Plant Mini Kit was used for the extraction of the DNA. The frozen powdered plant material was suspended in 3 ml of Buffer AP1 and 28 µl of RNAse A was added. After incubating at 65 °C, 910 µl of Buffer AP2 was added. The tubes were incubated on ice for 5 min and centrifuged thereafter to collect plant debris. Lysate (450 µl) was transferred to each of five to six QIAshredder tubes. The remainder of the DNA extraction steps were followed according to the kit protocol. The extracted DNA of each repetition was pooled after elution. DNA concentration was quantified using a Qubit 3.0 Fluorometer (Thermo Fisher Scientific) and visualized using a Labchip GX Touch 24 (PerkinElmer).

The extracted DNA was submitted to the Australian Genome Research Facility (AGRF) for library preparation and whole genome sequencing. The libraries for genome sequencing were prepared using the Illumina TruSeq Nano DNA HT Library Preparation Kit, according to the manufacturer’s instructions. Genomic DNA was sequenced using an Illumina HiSeqX sequencer with 150 bp paired-end technology at the Garvan Institute of Medical Research.

A total of 510485779 paired-end reads were sequenced. Based on previous flow cytometry analysis of two other Hydrocharitaceae members, *Najas minor* (2C=7.28) and *Eldodea Canadensis* (2C=7.54) (Hidalgo *et al.*, 2015), as well as genome size prediction (3628962593 bp, *k*=45) using the software Kmergenie (Chikhi and Medvedev, 2014) the sequencing coverage was estimated as ~40×. The sequences were deposited in a public repository (NCBI BioProject Accession PRJNA396090). Clones and low quality reads were removed using Sickle (Joshi and Fass, 2011).

### Pipeline to identify lost and conserved genes

The identification of lost and conserved genes was achieved using the mapping of whole genome shotgun sequencing reads against reference genomes based on a previous approach (Golicz *et al.*, 2015). The reads were mapped to coding sequences (CDS) of reference species using dc-megaBLAST (Camacho *et al.*, 2009) with e-value 1e−5. A custom python script, calculate_blast_coverage.py (downloadable at https://github.com/AppliedBioinformatics/H_ovalis_supplementary.git), was used to calculate the horizontal coverage of each CDS. The average coverage of each CDS across multiple reference species was calculated. If the average coverage was <2%, which means that mapped reads covered less than 2% of the length of a CDS, the orthologue was considered lost. If the average coverage was >50%, the orthologue was conserved.

### Orthologous gene cluster construction

A set of 16007 orthologous gene clusters (OGCs) conserved between seven model species with at least one gene originating from a monocot species, termed OGCsM (as defined in Table S1 in Golicz *et al.*, 2015), was used to represent orthologues highly conserved in plants.

Gene clusters unique to Zosteraceae were identified using all-against-all comparison with BLASTP (Camacho *et al.*, 2009) using the following parameters: ‘blastp -evalue 1e-5’, followed by OrthoMCL (Li *et al.*, 2003) between *Z. muelleri*, *Z. marina*, one dicot (Arabidopsis), and three other monocots (*Oryza sativa*, *Musa acuminata*, and *Spirodela polyrhiza*) (species selection based on Lee *et al.*, 2016). This group of orthologous genes was termed OGCZ.

### Lost and conserved *H. ovalis*, *Z. muelleri* and *Z. marina* genes in OGCsM

Primary transcript CDSs of five species (four land plants: Arabidopsis, *Oryza sativa*, *Musa acuminata*, and *Phoenix dactylifera*; one floating freshwater plant: *Spirodela polyrhiza*; versions as listed in Golicz *et al.*, 2015) were used as references for mapping of reads from *H. ovalis*. Presence and absence results from a previous publication were used for *Z. muelleri* and *Z. marina* (Golicz *et al.*, 2015). For each orthologue in OGCsM, lost or conserved status was assigned in each species.

### Lost and conserved *H. ovalis* genes in OGCZ

Primary transcript CDSs of *Z. muelleri* (http://www.appliedbioinformatics.com.au/index.php/Seagrass_Zmu_Genome; Lee *et al.*, 2016) and *Z. marina* (Phytozome 10; Olsen *et al.*, 2016) were used as references for *H. ovalis* read mapping. For each orthologue in OGCZ, lost or conserved status was assigned in *H. ovalis.*

### Gene ontology enrichment and word cloud plotting

GO annotation and enrichment were performed using the topGO package (Alexa and Rahnenfuhrer, 2010) based on a previous approach (Golicz *et al.*, 2015). OGCsM was used as background, except for the GO enrichment of OGCZ genes where Arabidopsis whole proteome (TAIR10) was used.

A word cloud was generated and coloured to represent the enriched significance of GO terms using the wordcloud package (Fellows, 2014).

### Inferring gene function through the level of protein domain conservation

OGCZ proteins of *Z. muelleri*, *Z. marina* and Arabidopsis were compared with TIGRFAM, ProDom, Panther, PfamA and PrositePatterns using InterProScan (version 5.14, Jones *et al.*, 2014) for motif and domain annotation. Domains of each protein were assigned with InterProScan IDs. The InterProScan IDs were compared between Arabidopsis and *Zosteraceae* genes for each OGCZ cluster.

### Assembly of *H. ovalis* protein and multiple sequence alignments with orthologues of other species

*Halophila ovalis* reads aligned to CDS of 50S ribosomal protein L16 were extracted and assembled using Spades v3.10.1 (Bankevich *et al.*, 2012) with the following commands: spades.py, only-assembler, 1 reads_1.fasta, 2 reads_2.fasta. Corresponding protein was aligned to the assembled contigs using Exonerate (Slater and Birney, 2005) with the following parameters: exonerate, model protein2genome, E 1, bestn 1, score 100, softmaskquery no, softmasktarget yes, minintron 20, maxintron 20000, ryo “>HAL_%qi_%qd\n%tas”. The aligned target regions were translated to protein sequences using the translate tool in ExPASy (Gasteiger *et al.*, 2003). Each *H. ovalis* protein sequence obtained was aligned with orthologues of selected species (Table S1 at *JXB* online) using MAFFT (Katoh *et al.*, 2002). A phylogenetic tree was plotted with PhyML (Guindon *et al.*, 2009) assuming the JTT model for amino acid substitution and gamma parameter for invariable sites (based on Huang *et al.*, 2016) using the alignments excluding the outgroup (charophyte and chlorophyte). The multiple-sequence alignments were visualized and coloured using Jalview (Waterhouse *et al.*, 2009).

## Results

### Read alignment of *H. ovalis* to reference species CDS

A total of 112202319 *H. ovalis* reads (10.9%) were discarded in the process of clonal removal and quality-based filtering (Table S2). Out of the remaining 908769239 *H. ovalis* reads, 2.7% (24495631) aligned to Arabidopsis CDS, 5.6% (50565060) aligned to *Oryza sativa* CDS, 1.3% (11617255) aligned to *Musa acuminata* CDS, 0.8% (7367361) aligned to *Phoenix dactylifera* CDS and 1.8% (16600802) aligned to *Spirodela polyrhiza* CDS. For the seagrass reference species, 1.8% (16727940) and 0.5% (5005993) of *H. ovalis* reads aligned to *Z. muelleri* and *Z. marina* CDSs, respectively.

### Conservation of core biological processes

A total of 4367 OGCsM genes, out of 16007, were conserved in *H. ovalis*. When compared with conserved genes previously described in *Z. muelleri* and *Z. marina* (Golicz *et al.*, 2015; Lee *et al.*, 2016; Olsen *et al.*, 2016), 3335 (76.4%) genes were conserved in all three seagrass species, 377 genes were shared with either *Z. muelleri* or *Z. marina*, and 655 genes were only conserved in *H. ovalis*. A total of 508 genes were only conserved in the Zosteraceae species. A full list of genes conserved in *H. ovalis* and their presence in other seagrass species is presented in Table S3. The GO terms enriched in these 4367 OGCsM genes conserved in *H. ovalis* involved core biological pathways such as photosynthesis, chlorophyll biosynthesis, and glycolytic processes, as well as response to stresses such as cadmium (Table 1).

**Table 1.** Significantly enriched biological process GO terms in the genes conserved in *H. ovalis* compared with five other plant species (Arabidopsis, *Oryza sativa*, *Musa acuminata*, *Phoenix dactylifera*, and *Spirodela polyrhiza*)

GO ID	Term	*P* value
GO:0046686	Response to cadmium ion	3.0 × 10^−30^
GO:0006412	Translation	4.1 × 10^−28^
GO:0046496	Nicotinamide nucleotide metabolic process	3.9 × 10^−16^
GO:0006099	Tricarboxylic acid cycle	1.7 × 10^−13^
GO:0015991	ATP hydrolysis-coupled proton transport	1.0 × 10^−12^
GO:1901566	Organonitrogen compound biosynthetic process	7.9 × 10^−11^
GO:0043039	tRNA aminoacylation	1.2 × 10^−10^
GO:0006090	Pyruvate metabolic process	1.9 × 10^−10^
GO:1901293	Nucleoside phosphate biosynthetic process	2.3 × 10^−10^
GO:0009156	Ribonucleoside monophosphate biosynthetic process	2.7 × 10^−10^
GO:0009225	Nucleotide-sugar metabolic process	5.4 × 10^−10^
GO:0007264	Small GTPase-mediated signal transduction	8.2 × 10^−9^
GO:0046034	ATP metabolic process	8.4 × 10^−9^
GO:0006108	Malate metabolic process	1.4 × 10^−8^
GO:0006006	Glucose metabolic process	1.8 × 10^−8^
GO:0034622	Cellular macromolecular complex assembly	2.3 × 10^−8^
GO:0071702	Organic substance transport	2.4 × 10^−8^
GO:0018105	Peptidyl-serine phosphorylation	3.2 × 10^−8^
GO:0009250	Glucan biosynthetic process	2.0 × 10^−7^
GO:0016192	Vesicle-mediated transport	2.0 × 10^−7^
GO:0010499	Proteasomal ubiquitin-independent protein catabolic process	3.4 × 10^−7^
GO:0043094	Cellular metabolic compound salvage	6.7 × 10^−7^
GO:0015994	Chlorophyll metabolic process	1.2 × 10^−6^
GO:0034613	Cellular protein localization	1.4 × 10^−6^
GO:0006536	Glutamate metabolic process	1.9 × 10^−6^
GO:0005985	Sucrose metabolic process	5.3 × 10^−6^
GO:0098656	Anion transmembrane transport	5.7 × 10^−6^
GO:0015672	Monovalent inorganic cation transport	7.5 × 10^−6^
GO:0009932	Cell tip growth	9.9 × 10^−6^
GO:0006081	Cellular aldehyde metabolic process	1.0 × 10^−5^
GO:0018298	Protein–chromophore linkage	1.0 × 10^−5^
GO:0030163	Protein catabolic process	1.3 × 10^−5^
GO:0048588	Developmental cell growth	1.3 × 10^−5^
GO:0006102	Isocitrate metabolic process	2.4 × 10^−5^
GO:0006607	NLS-bearing protein import into nucleus	2.5 × 10^−5^
GO:0015977	Carbon fixation	5.1 × 10^−5^
GO:0015979	Photosynthesis	5.4 × 10^−5^
GO:0006563	L-Serine metabolic process	6.0 × 10^−5^
GO:0006268	DNA unwinding involved in DNA replication	6.9 × 10^−5^
GO:0007035	Vacuolar acidification	6.9 × 10^−5^
GO:0009768	Photosynthesis, light harvesting in photosystem I	7.2 × 10^−5^
GO:0016197	Endosomal transport	8.1 × 10^−5^
GO:0030048	Actin filament-based movement	0.00012
GO:0009651	Response to salt stress	0.00012
GO:0006206	Pyrimidine nucleobase metabolic process	0.00013
GO:0030243	Cellulose metabolic process	0.00014
GO:0097164	Ammonium ion metabolic process	0.00015
GO:0010315	Auxin efflux	0.00015
GO:0006551	Leucine metabolic process	0.00017
GO:0006085	Acetyl-CoA biosynthetic process	0.00018
GO:0045899	Positive regulation of RNA polymerase II transcriptional preinitiation complex assembly	0.00020
GO:0032012	Regulation of ARF protein signal transduction	0.00020
GO:0009735	Response to cytokinin	0.00027
GO:0006782	Protoporphyrinogen IX biosynthetic process	0.00030
GO:0009846	Pollen germination	0.00032
GO:1901679	Nucleotide transmembrane transport	0.00041
GO:0030042	Actin filament depolymerization	0.00048
GO:0006558	L-Phenylalanine metabolic process	0.00050
GO:0006544	Glycine metabolic process	0.00057
GO:0035999	Tetrahydrofolate interconversion	0.00096
GO:0009066	Aspartate family amino acid metabolic process	0.00103
GO:0006222	UMP biosynthetic process	0.00113
GO:0046500	*S*-Adenosylmethionine metabolic process	0.00125
GO:0015804	Neutral amino acid transport	0.00125
GO:0006097	Glyoxylate cycle	0.00125
GO:0030433	Endoplasmic reticulum-associated ubiquitin- dependent protein degradation	0.00132
GO:0019627	Urea metabolic process	0.00169
GO:0015800	Acidic amino acid transport	0.00169
GO:0000398	mRNA splicing, via spliceosome	0.00170
GO:0010540	Basipetal auxin transport	0.00235
GO:0035435	Phosphate ion transmembrane transport	0.00258
GO:0006457	Protein folding	0.00262
GO:0051259	Protein oligomerization	0.00265
GO:0006525	Arginine metabolic process	0.00265
GO:0016482	Cytoplasmic transport	0.00284
GO:0016036	Cellular response to phosphate starvation	0.00288
GO:0043604	Amide biosynthetic process	0.00295
GO:0019395	Fatty acid oxidation	0.00317
GO:0006570	Tyrosine metabolic process	0.00326
GO:0052646	Alditol phosphate metabolic process	0.00326
GO:0010043	Response to zinc ion	0.00346
GO:0055085	Transmembrane transport	0.00377
GO:0000338	Protein deneddylation	0.00436
GO:0006002	Fructose 6-phosphate metabolic process	0.00436
GO:0043650	Dicarboxylic acid biosynthetic process	0.00439
GO:0010501	RNA secondary structure unwinding	0.00439
GO:0009629	Response to gravity	0.00447
GO:0006103	2-Oxoglutarate metabolic process	0.00490
GO:0009833	Plant-type primary cell wall biogenesis	0.00490
GO:0006610	Ribosomal protein import into nucleus	0.00490
GO:0010541	Acropetal auxin transport	0.00540
GO:0006012	Galactose metabolic process	0.00540
GO:0006511	Ubiquitin-dependent protein catabolic process	0.00630
GO:0051275	β-Glucan catabolic process	0.00735
GO:0009141	Nucleoside triphosphate metabolic process	0.00770
GO:0018208	Peptidyl-proline modification	0.00803
GO:0006486	Protein glycosylation	0.00804
GO:0009624	Response to nematode	0.00847
GO:1904659	Glucose transmembrane transport	0.00872
GO:0046323	Glucose import	0.00872
GO:0006631	Fatty acid metabolic process	0.00878
GO:0044282	Small molecule catabolic process	0.00886

### Gene loss in *H. ovalis* and comparison of lost genes between the three seagrass species

A total of 1822 OGCsM genes were lost in *H. ovalis*, and these were compared with those previously reported as lost in both *Z. muelleri* and *Z. marina* (Golicz *et al.*, 2015; Lee *et al.*, 2016; Olsen *et al.*, 2016) (Table S4). A total of 1197 (65.6%) lost genes were shared between all three seagrass species, 187 were shared with either *Z. muelleri* or *Z. marina*, and 412 were only lost in *H. ovalis*. In comparison, 743 genes were only lost in the Zosteraceae lineage. Enriched GO terms for the 1822 OGCsM genes highlighted the loss of genes associated with ethylene synthesis and perception, and stomatal development (Table 2). The presence or absence of genes involved in stomatal development, ethylene synthesis and signalling, and terpenoid biosynthesis in *H. ovalis*, *Z. marina*, and *Z. muelleri* are listed in Table 3.

**Table 2.** Significantly enriched biological process GO terms in the genes conserved in five other plant species (Arabidopsis, *Oryza sativa*, *Musa acuminata*, *Phoenix dactylifera* and *Spirodela polyrhiza*) but absent in *H. ovalis*

Function	GO ID	Term	*P* value
Ethylene synthesis and signalling	GO:0009835	Fruit ripening	4.1 × 10^−10^
	GO:0042218	1-Aminocyclopropane-1-carboxylate biosynthetic process	6.3 × 10^−10^
	GO:0009693	Ethylene biosynthetic process	1.9 × 10^−8^
	GO:0010105	Negative regulation of ethylene-activated signalling pathway	1.2 × 10^−6^
Stomata development	GO:0010375	Stomatal complex patterning	0.00012
	GO:2000038	Regulation of stomatal complex development	0.00608
Others	GO:0045168	Cell–cell signalling involved in cell fate commitment	3.1 × 10^−6^
	GO:0006952	Defence response	9.4 × 10^−6^
	GO:0009626	Plant-type hypersensitive response	1.0 × 10^−5^
	GO:0031640	Killing of cells of other organism	8.4 × 10^−5^
	GO:0010039	Response to iron ion	0.00011
	GO:0034644	Cellular response to UV	0.00014
	GO:0071484	Cellular response to light intensity	0.00018
	GO:0009773	Photosynthetic electron transport in photosystem I	0.00023
	GO:0080027	Response to herbivore	0.00034
	GO:0033473	Indoleacetic acid conjugate metabolic process	0.00034
	GO:0009696	Salicylic acid metabolic process	0.00059
	GO:0033609	Oxalate metabolic process	0.00093
	GO:0050832	Defence response to fungus	0.00113
	GO:0071423	Malate transmembrane transport	0.00209
	GO:0042542	Response to hydrogen peroxide	0.00234
	GO:1900426	Positive regulation of defence response to bacterium	0.00239
	GO:0010876	Lipid localization	0.00313
	GO:0018106	Peptidyl-histidine phosphorylation	0.00404
	GO:0046688	Response to copper ion	0.00558
	GO:0010257	NADH dehydrogenase complex assembly	0.00608
	GO:0009838	Abscission	0.00704
	GO:0071732	Cellular response to nitric oxide	0.00791

**Table 3.** Presence and absence of genes involved in stomatal development, ethylene synthesis and signalling, and terpenoid biosynthesis in OGCsM, *H. ovalis*, *Z. marina*, and *Z. muelleri*

Gene ID	Protein name	Function	Conserved in OGCsM	Presence in *H. ovalis*	Presence in *Z. muelleri*	Presence in *Z. marina*
Stomata development						
*AT1G04110*	SBT1.2	Spacing and patterning	+	NA	−	−
*AT4G12970*	EPFL9	Spacing and patterning	+	−	−	−
*AT2G20875*	EPF1	Spacing and patterning	+	−	−	−
*AT1G80080*	TMM	Spacing and patterning	+	−	−	−
*AT1G34245*	EPF2	Spacing and patterning	+	−	−	−
*AT2G02820*	MYB88	Differentiation	−	NA	NA	−
*AT3G06120*	MUTE	Differentiation	+	−	−	−
*AT5G53210*	SPCH	Differentiation	+	−	−	−
*AT3G24140*	FAMA	Differentiation	+	NA	NA	−
*AT1G12860*	SCRM2	Differentiation	−	NA	NA	−
*AT1G14350*	FLP	Differentiation	+	−	−	−
Ethylene synthesis and signalling						
*AT2G19590*	ACO1	ACC oxidase	+	−	−	−
*AT1G62380*	ACO2	ACC oxidase	+	−	−	−
*AT1G05010*	ACO4	ACC oxidase	+	−	−	−
*AT1G77330*	ACO5	ACC oxidase	+	−	−	−
*AT3G61510*	ACS1	ACC synthase	+	−	−	−
*AT1G01480*	ACS2	ACC synthase	+	−	−	−
*AT2G22810*	ACS4	ACC synthase	+	−	−	−
*AT5G65800*	ACS5	ACC synthase	+	−	−	−
*AT4G11280*	ACS6	ACC synthase	+	−	−	−
*AT4G26200*	ACS7	ACC synthase	+	−	−	−
*AT4G37770*	ACS8	ACC synthase	+	−	−	−
*AT3G49700*	ACS9	ACC synthase	+	−	−	−
*AT4G08040*	ACS11	ACC synthase	+	−	−	−
*AT2G40940*	ERS1	Ethylene receptor	+	−	−	−
*AT1G66340*	ETR1	Ethylene receptor	+	−	−	−
*AT3G23150*	ETR2	Ethylene receptor	+	−	−	−
*AT3G04580*	EIN4	Ethylene receptor	+	−	−	−
*AT5G03730*	CTR1	Raf-like kinase	+	NA	NA	−
*AT5G03280*	EIN2	Signal transducer	+	NA	−	−
*AT2G25490*	EBF1	EIN2 degradation	+	−	−	−
*AT5G25350*	EBF2	EIN2 degradation	+	−	−	−
Terpenoid biosynthesis						
*AT3G25820*	TPS-CIN	Terpene synthase	+	−	−	−
*AT3G25830*	TPS23	Terpene synthase	+	−	−	−
*AT4G16740*	TPS03	Terpene synthase	−	−	−	−
*AT2G24210*	TPS10	Terpene synthase	+	−	−	−
*AT3G25810*	TPS24	Terpene synthase	−	−	−	−

Categories are: gene present (+), gene absent (−), and information not available (NA).

### *Halophila ovalis* lost genes encoding NADH dehydrogenase-like complex assembly

The five most significantly enriched GO terms in the 412 genes that were only lost in *H. ovalis* were cellular response to light intensity (GO:0071484), cellular response to UV (GO:0034644), photosynthetic electron transport in photosystem I (GO:0009773), NADH dehydrogenase complex assembly (GO:0010257), and cellular response to salt stress (GO:0071472). A complete list of all significantly enriched terms is given in Table S5. Closer examination revealed the loss of 23 (15 nuclear and 8 chloroplast) genes that encode the five subcomplexes in the NDH complex (Table 4). In addition, 17 genes required for the supercomplex formation, including tethering of NDH to photosystem I, assembly of subunits, accessory proteins, and transcription factors, were absent in *H. ovalis*. Two proteins required for nitrate uptake and assimilation, nitrogen reductase 1 (NR1) and nitrate transporter (NRT3.1), were also lost in *H. ovalis.*

**Table 4.** Presence and absence of 40 nuclear and chloroplast-encoded genes involved in formation of the NDH complex

Gene ID	Protein name	Function	Presence in OGCsM	Presence in *H. ovalis*	Presence in *Z. muelleri*	Presence in *Z. marina*
Nuclear encoded						
*AT1G70760*	NDHL	Subunit A	+	NA	+	+
*AT4G37925*	NDHM	Subunit A	+	−	+	+
*AT5G58260*	NDHN	Subunit A	+	NA	+	+
*AT1G74880*	NDHO	Subunit A	+	−	+	+
*AT4G23890*	NDHS	Subunit ED	+	−	NA	+
*AT4G09350*	NDHT	Subunit ED	+	−	+	+
*AT5G21430*	NDHU	Subunit ED	+	−	+	+
*AT1G15980*	PNSB1	Subunit B	+	−	NA	+
*AT1G64770*	PNSB2	Subunit B	+	−	+	+
*AT3G16250*	PNSB3	Subunit B	+	−	+	+
*AT1G18730*	PNSB4	Subunit B	+	−	+	+
*AT2G39470*	PNSL1	Subunit B	+	−	+	+
*AT1G14150*	PNSL2	Subunit L	+	−	+	+
*AT3G01440*	PNSL3	Subunit L	+	NA	+	+
*AT4G39710*	PNSL4	Subunit L	+	−	+	+
*AT5G13120*	PNSL5	Subunit L	+	+	+	+
*AT2G47910*	CRR6	Complex formation	+	−	+	+
*AT5G39210*	CRR7	Complex formation	+	−	+	+
*AT1G45474*	Lhca5	Complex formation	+	−	+	+
*AT1G19150*	Lhca6	Complex formation	+	NA	+	+
*AT1G26230*	CRR27	Complex formation	+	NA	+	+
*AT1G51100*	CRR41	Complex formation	+	NA	+	+
*AT2G05620*	PGR5	Proton gradient regulation	+	NA	+	+
*AT4G22890*	PGRL1A	Proton gradient regulation	+	+	+	+
*AT3G46790*	CRR2	Unknown	+	NA	+	+
*AT2G01590*	CRR3	Unknown	+	−	−	+
*AT5G20935*	CRR42	Unknown	+	−	+	+
*AT2G01918*	PQL3	Unknown	+	−	−	+
*AT1G55370*	NDF5	Unknown	+	−	+	+
Chloroplast encoded						
*ATCG00890* *ATCG01250*	NDHB	Subunit M	+	NA	+	+
*ATCG01010*	NDHF	Subunit M	+	NA	+	+
*ATCG00440*	NDHC	Subunit M	+	−	+	+
*ATCG01050*	NDHD	Subunit M	+	−	+	+
*ATCG01070*	NDHE	Subunit M	+	−	+	+
*ATCG01100*	NDHA	Subunit M	+	−	+	+
*ATCG01080*	NDHG	Subunit M	+	NA	+	+
*ATCG01110*	NDHH	Subunit A	+	+	+	+
*ATCG00420*	NDHJ	Subunit A	+	−	+	+
*ATCG00430*	NDHK	Subunit A	+	−	+	+
*ATCG01090*	NDHI	Subunit A	+	−	+	+

Categories are: gene present (+), gene absent (−), and information not available (NA).

### Co-evolution of genes for intracellular transport, cell wall, and ion transport-related genes in *H. ovalis* and *Zostera*

We identified a set of 1748 genes that are unique to *Z. muelleri* and *Z. marina*, termed OGCZ (Fig. S1; Table S6), with 57 also found to be conserved in *H. ovalis* (Table S7). Putative functions of these 57 genes were inferred by matching their protein domains to the corresponding best aligned Arabidopsis gene. Out of 57 OGCZ groups, 45 have identical domains (indicated as InterProScan IDs) to the corresponding Arabidopsis genes. A total of eight of them have fewer or different domains from Arabidopsis, and four of them have no domains assigned (Table S7).

**Fig. 1.**
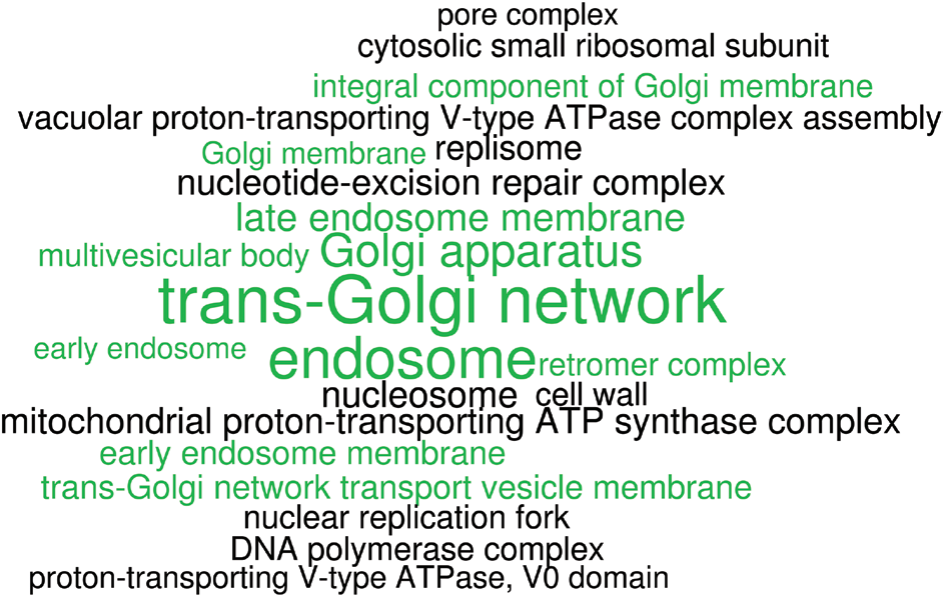
Significantly enriched cellular component GO terms in seagrass-specific genes. Terms in green are subcomponents or organelles of the intracellular transport pathways.

The majority of the 57 OGCZ genes conserved in *H. ovalis* are predicted to be involved in protein secretion and intracellular transport, with significantly enriched terms annotated with cellular component ontology, including organelles of the intracellular transport pathways, namely Golgi apparatus, trans-Golgi network, and endosome, and nearly half of the remaining terms intracellular transport-related (Fig. 1). A total of 13 genes are predicted to function in protein secretion and intracellular transport, mainly as transport proteins or transport regulators. Nine genes are associated with cell wall construction, organization, and modification, while other predicted functions include ion or proton transport, lipid catabolism, transcription and translation-related, protein ubiquitination, and histone assembly (Table 5).

**Table 5.** Fifty-seven orthologous groups of seagrass-specific genes shared in two Zosteraceae species (*Z. muelleri* and *Z. marina*) and *Halophila* categorized by predicted function

Category of related function	Name of best TAIR10 hit corresponding to *Zostera* orthologue	ID of best TAIR10 hit corresponding to *Zostera* orthologue	Putative gene function
Protein secretion and intracellular transport	Endoplasmic reticulum retention defective 2B	AT3G25040.1	Retention mechanism
	Endoplasmic reticulum-type calcium-transporting ATPase 3	AT1G10130.1	Calcium and manganese ion transport
	RAB GTPase homologue A1F	AT5G60860.1	GTPase activity
	RAB GTPase homologue A2B	AT1G07410.1	GTPase activity
	Secretory carrier 3	AT1G61250.1	Integral membrane protein
	NOD26-like intrinsic protein 1;2	AT4G18910.1	Aquaporin
	Mitochondrial substrate carrier family protein	AT3G53940.1	Substrate transport
	Mitochondrial import inner membrane translocase subunit Tim17/Tim22/Tim23 family protein	AT5G63000.1	Protein transport
	Transducin/WD40 repeat-like superfamily protein	AT3G01340.1	Protein transport
	Protein of unknown function	AT1G09330.1	—
Cell wall	Expansin A16	AT3G55500.1	Cell wall loosening
	Expansin A1	AT1G69530.2	Cell wall loosening
	Galacturonosyltransferase-like 2	AT3G50760.1	Cell wall organization
	Xyloglucan endotransglucosylase/hydrolase 5	AT5G13870.1	Cell wall organization
	Glucan synthase-like 8	AT2G36850.1	Callose synthesis
	*S*-Adenosyl-L-methionine-dependent methyltransferases superfamily protein	AT4G34050.1	Lignin biosynthesis
	Peroxidase superfamily protein	AT5G05340.1	Lignin biosynthesis
	Cotton Golgi-related 2 (pectin methyltransferase)	AT3G49720.1	Cell wall modification
	Vascular related NAC-domain protein 1	AT2G18060.1	Xylem secondary cell wall formation
Ion flux and sequestering	ATP synthase epsilon chain, mitochondrial	AT1G51650.1	Proton-transporting ATPase activity
	Vacuolar proton ATPase A1	AT2G28520.1	Proton-transporting ATPase activity
	Calmodulin 4	AT1G66410.1	Calcium ion binding
Lipid catabolism	Trigalactosyldiacylglycerol 5	AT1G27695.1	Lipid transport
	GDSL-like lipase/acylhydrolase superfamily protein	AT1G29670.1 AT5G45670.1	Lipid catabolic process
	Peroxin 6	AT1G03000.1	Peroxisomal matrix protein import
	Alkaline phytoceramidase	AT4G22330.1	Ceramide synthase involved in sphingolipid metabolism
Transcription-related	RNA polymerase subunit beta	ATCG00190.1	Constituent of RNA polymerase B
	Pre-mRNA-splicing factor SPF27 homologue	AT3G18165.1	mRNA splicing of resistance genes
Ribosome/ translation-related	Ribosomal protein L16	ATCG00790.1	Structural constituent of ribosome
	Ribosomal protein S26e family protein	AT2G40510.1	Structural constituent of ribosome
	Ribosomal protein S8e family protein	AT5G59240.1	Structural constituent of ribosome
	Ribosomal protein S2	ATCG00160.1	Structural constituent of ribosome
	Eukaryotic translation initiation factor 3A	AT4G11420.1	Constituent of eukaryotic initiation factor 3
Protein ubiquitination	F-box protein PP2-A13	AT3G61060.1	Protein ubiquitination
	BTB/POZ domain-containing protein	AT1G63850.1	Protein ubiquitination
	Ubiquitin-conjugating enzyme 28	AT1G64230.1	Protein ubiquitination
	Ubiquitin-like protein 5	AT5G42300.1	Ubiquitin-like modification
Histone	Histone H2A.2	AT3G20670.1	Histones/DNA binding/nucleosome assembly
	Histone H3.3	AT4G40030.2	Histones/DNA binding/nucleosome assembly
Others	Photosystem II light harvesting complex gene 2.1	AT2G05100.1	Constituent of light harvesting complex II
	Alternative oxidase 1A	AT3G22370.1	Alternative oxidase activity
	Tubulin folding cofactor D	AT3G60740.1	Microtubule stability
	Asparagine synthetase 2	AT5G65010.2	Asparagine biosynthesis
	Glutamate-1-semialdehyde 2,1-aminomutase 2	AT3G48730.1	Porphyrin-containing compound metabolism
	Membrane-associated progesterone binding protein 3	AT3G48890.1	Porphyrin binding
	Thioredoxin superfamily protein	AT3G62950.1	Electron carrier activity
	DNA polymerase epsilon catalytic subunit	AT1G08260.1	DNA replication proofreading
	NAC domain containing protein 32	AT1G77450.1	Transcription factor
	DNA-binding protein phosphatase 1	AT2G25620.1	Protein phosphatase activity
	Protein kinase 1B	AT2G28930.1	Serine/threonine kinase activity
	UDP-glycosyltransferase superfamily protein	AT5G04480.1	—
	Adenine nucleotide alpha hydrolases-like superfamily protein	AT1G11360.4	—
	Protein of unknown function (DUF300)	AT1G11200.1	—
	Protein of unknown function (DUF803)	AT1G34470.1	—

Gene functions were predicted with corresponding Arabidopsis gene of highest sequence similarity.

### Molecular comparison of seagrass ribosomal proteins

**Fig. 2.**
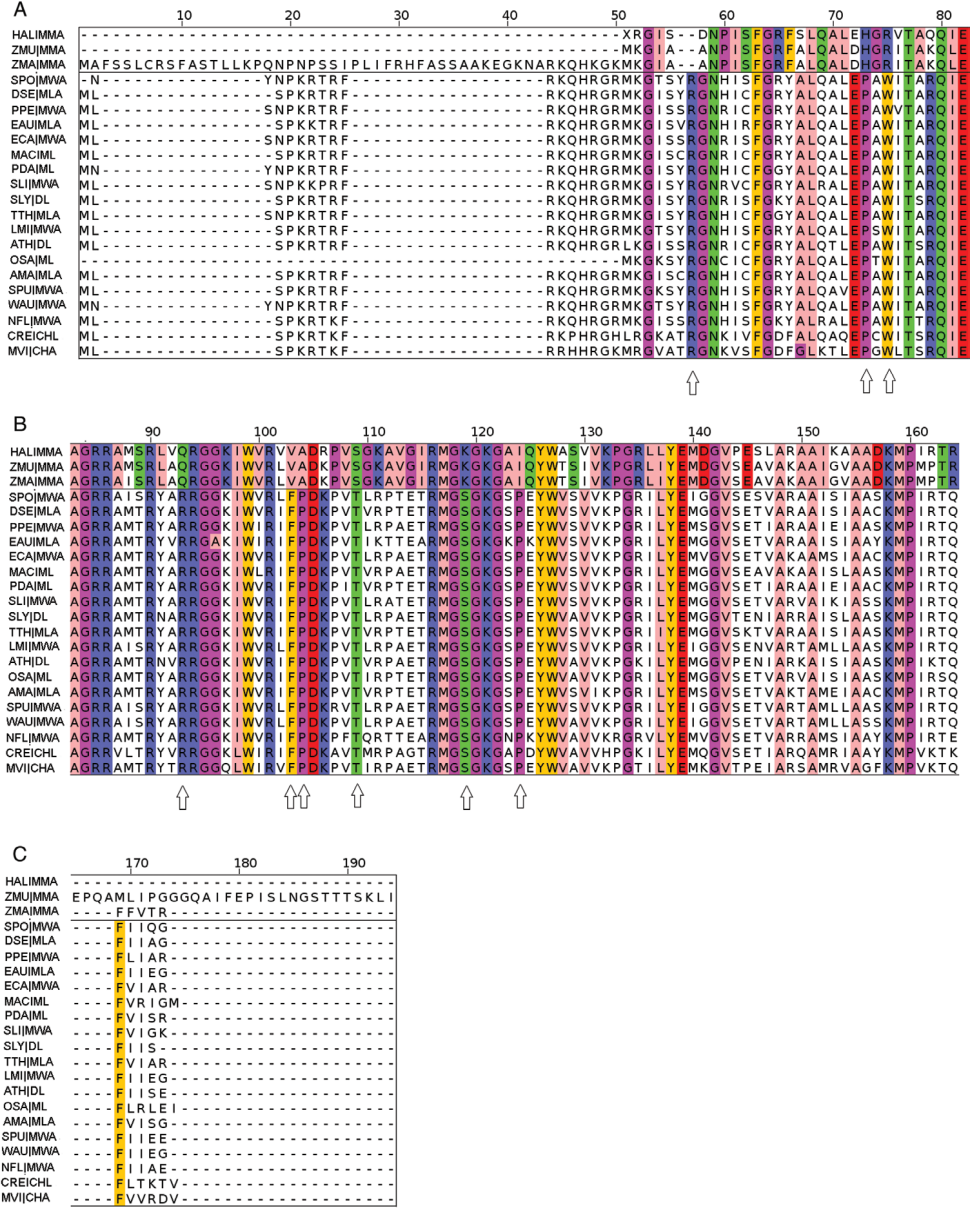
Ribosomal protein L16 multiple sequence alignments between 19 species (AMA, *Alocasia macrorhizzos*; ATH, Arabidopsis; DSE, *Dieffenbachia seguine*; EAU, *Epiprenum aureum*; ECA, *Elodia canadensis*; LMI, *Lemna minor*; MAC, *Musa acuminata*; NFL, *Najas flexilis*; OSA, *Oryza sativa*; PDA, *Phoenix dactylifera*; PPE, *Potamogeton perfoliatus*; SLI, *Sagittaria lichuanensis*; SLY, *Solanum lycopersicum*; SPO, *Spirodela polyrhiza*; SPU, *Spirodela pundata*; TTH, *Tofieldia thibetica*; WAU, *Wolfia australiana*) together with three seagrasses (HAL, *H. ovalis*; ZMA, *Z. marina*; ZMU, *Z. muelleri*). Species and corresponding IDs are listed in Table S1. Amino acids that were conserved within the non-seagrass group or among seagrasses are coloured according to physicochemical properties based on ‘Zappo’ colour scheme. White arrows indicated seagrass-specific mutations.

Ribosomal 50S L16 orthologues from the two *Zostera* species, *H. ovalis*, and 12 species in the Alismatales order were aligned, together with predicted proteins from five model land plants (Table S1). We identified nine amino acid positions that appear to be specific to the seagrasses *H. ovalis*, *Z. muelleri*, and *Z. marina* (white arrows in Fig. 2) and conserved among the other 17 angiosperms (12 belong to the Alismatales order, eight are freshwater plants), one charophyte and one chlorophyte.

**Fig. 3.**
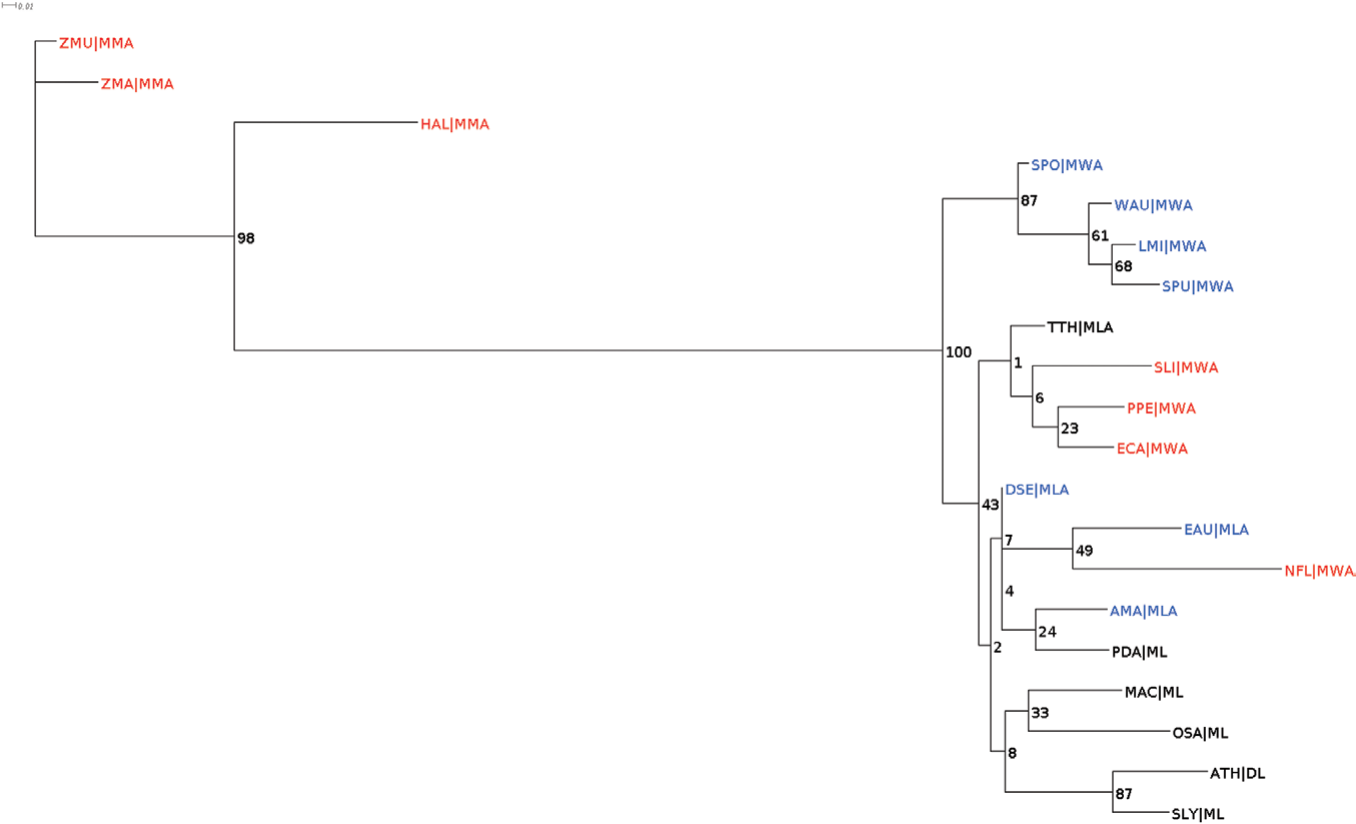
Phylogenetic tree showing distance between ribosome protein L16 sequences of 17 species (AMA, *Alocasia macrorhizzos*; ATH, Arabidopsis; DSE, *Dieffenbachia seguine*; EAU, *Epiprenum aureum*; ECA, *Elodia canadensis*; LMI, *Lemna minor*; MAC, *Musa acuminata*; NFL, *Najas flexilis*; OSA, *Oryza sativa*; PDA, *Phoenix dactylifera*; PPE, *Potamogeton perfoliatus*; SLI, *Sagittaria lichuanensis*; SLY, *Solanum lycopersicum*; SPO, *Spirodela polyrhiza*; SPU, *Spirodela pundata*; TTH, *Tofieldia thibetica*; WAU, *Wolfia australiana*) together with three seagrasses (HAL, *H. ovalis*; ZMA, *Z. marina*; ZMU, *Z. muelleri*). The order and habitat of species were indicated in the second part of each ID: DL, dicot, land; ML, monocot, land; MLA, monocot, land, Alismatales; MMA, monocot, marine, Alismatales; MWA, monocot, freshwater, Alismatales. Complete details are listed in Table S1. IDs coloured in red are members of core Alismatids, blue are members of Araceae, and black are others. Branches are labelled with bootstrap values (%).

A phylogenetic tree for these 22 species based on this protein sequence, which describes the relationships between orthologues of these 22 species, separates the seagrass species (*H. ovalis*, *Z. muelleri*, and *Z. marina*) from the other species (Fig. 3). The separation of the two *Zostera* orthologues from *H. ovalis* is also well-supported. Sister genera of both *Halophila* and *Zostera* grouped together as members of core alismatids (red in Fig. 3) (Alismatidae *sensu*; Les and Tippery, 2013).

## Discussion

The concurrent absence of multiple genes in *H. ovalis*, *Z. muelleri*, and *Z. marina* suggests independently evolved convergent adaptation of seagrasses to the marine environment. Seagrass leaves lack stomata and the flowers have simplified structures when compared with terrestrial angiosperms (Kuo and Hartog, 2006). The loss of genes in stomata patterning and differentiation, and in sepal and petal development was previously described in *Zostera*, together with the loss of gaseous hormones and metabolites, such as ethylene, methyl jasmonate, and secondary volatile terpenes (Golicz *et al.*, 2015; Lee *et al.*, 2016; Olsen *et al.*, 2016). Consistent with the low diffusion rate of gases underwater, the absence of ethylene production in seagrasses avoids accumulation in the tissues. In flood-adapted land plants, the ethylene signal is used to sense submergence and induces a response to flooding (Voesenek *et al.*, 2015). Ethylene biosynthesis and signalling also play an important role in plant response to salinity (Zhang *et al.*, 2016). There is conflicting evidence of ethylene as a positive or negative regulator during salinity stress in different species at different developmental stages (Tao *et al.*, 2015), suggesting that some species adjust their sensitivity to environmental factors through regulation of the ethylene signalling pathway. As the loss of ethylene genes is observed in both *H. ovalis* and *Zostera*, it is likely that ethylene is selected against during seagrass adaptation to a submerged marine lifestyle.

The sharing of OGCZ genes in *H. ovalis* to form a seagrass-specific gene set identifies orthologous relationships that appear to be unique to seagrass adaptation, despite their evolutionary distance and multiple origins. It is important to note that these 57 OGCZ genes are not novel genes but genes that have diverged sufficiently to cluster separately from other plants in orthologue analysis. The functions of these genes were not annotated but inferred by homology using annotated Arabidopsis genes. The majority of these genes are predicted to be involved in intracellular transport and in cell wall organization and modification. In plant cells, secreted proteins are processed through the Golgi apparatus as cargo molecules and sorted by receptors in the trans-Golgi network to different destinations (Brandizzi and Barlowe, 2013). Non-cellulosic cell wall matrix polysaccharides are among the wide range of vesicles synthesized and transported by the Golgi apparatus (Driouich *et al.*, 1993; Lerouxel *et al.*, 2006; Driouich *et al.*, 2012). Besides catalytic mechanisms of glycosyltransferases and nucleotide-sugar conversions for polysaccharide assembly, the Golgi is also responsible for methylation of the cell wall polysaccharides. There are significant differences between cell walls of seagrasses and land plants. Seagrass cell walls contain sulfated polysaccharides (Aquino *et al.*, 2005) and seagrass pectin contains a rare class of apiose-substituted homogalacturonan (Ovodov *et al.*, 1971) with low levels of methyl esterification (Khotimchenko *et al.*, 2012). These two modifications are thought to provide salt tolerance by increasing the polyanionic potential of cell walls (Aquino *et al.*, 2005, 2011; Olsen *et al.*, 2016). An expansion of pectin catabolic and methylesterase genes was observed in the genomes of *Z. muelleri* (Lee *et al.*, 2016) and *Z. marina* (Olsen *et al.*, 2016), suggesting complex pectin modification in seagrasses. Interestingly, within the list of seagrass-specific genes conserved in *H. ovalis*, CGR2 (cotton Golgi-related 2), a methyltransferase, was shown to be involved in pectin methylesterification in Arabidopsis (Weraduwage *et al.*, 2016). Tubulin cofactor, which is responsible for the stability of microtubules (Zhu *et al.*, 2015), is also found to be conserved among seagrasses. A total of five genes that encode RAB GTPases, the key regulators of vesicle trafficking (Miserey-Lenkei *et al.*, 2010; Valente *et al.*, 2010), were also conserved across both seagrass lineages. In Arabidopsis, knockouts of some members of the RAB GTPases have demonstrated roles in salinity stress tolerance (Asaoka *et al.*, 2013). It is likely that this conservation of cell wall-related genes, as well as proteins involved in intracellular transport, in both families of seagrasses is linked to modification of cell wall composition as one of the adaptations to osmotic stress.

Multiple salt-tolerance mechanisms have been hypothesized in seagrasses (reviewed in Touchette, 2007), including cell wall rigidity, selective ion flux and vacuolar ion sequestering, and the synthesis of compatible solutes and amino acids (Ye and Zhao, 2003; Carpaneto *et al.*, 2004; Touchette *et al.*, 2014; Cambridge *et al.*, 2017). To avoid salt damage, plant cells adjust osmotic balance through influx and efflux of ions through the transmembrane transport proteins, assisted by H^+^ pumps (Hasegawa, 2013). Three genes, namely a component of a vacuolar proton pump, ATP synthase and calmodulin, were identified as conserved across the two seagrass lineages. Moreover, vacuolar proton ATPase A1 has been shown to be responsive to salt stress in sugar beet (Kirsch *et al.*, 1996). This collection of genes may have a role in osmotic homeostasis of cells in the marine environment.

Lipid transport and catabolism is another important role of the intracellular transport system. The endoplasmic reticulum synthesizes and exports phospholipids, sterols, and storage lipids for various purposes, including formation of membrane structures (van Meer *et al.*, 2008). A total of four genes involved in lipid transport and catabolism were conserved in all three seagrass species, including ceramidase, which is responsible for sphingolipid metabolism. Sphingolipids provide membrane structure and are involved in cellular signal transduction (Hannun and Obeid, 2008). The difference between lipids of seagrasses and land plants is not well understood, but expansion in genes related to sphingolipid metabolism was observed in *Z. marina* when compared with duckweed (Olsen *et al.*, 2016). Another alkaline ceramidase had been shown to regulate cell turgor pressure in Arabidopsis (Chen *et al.*, 2015), but more evidence is needed to determine whether seagrass-specific lipid metabolism plays a role in marine adaptation.

Two members of the core histone family are conserved in seagrasses. The domains in histone families, particularly H2A and H3, demonstrate expansion in numbers and variety, but with strong conservation of each variant across species (Kawashima *et al.*, 2015). Ribosomal constituents were previously identified as modified in *Z. muelleri* when compared with land plants (Lee *et al.*, 2016) and positively selected in *Z. marina* and *P. oceanica* (Wissler *et al.*, 2011), and our results demonstrate that these genes are also conserved in *H. ovalis*. The basis for the observed differences in ribosomal gene sequences is not known, but it is postulated to be related to salt tolerance. Translation, and consequently protein synthesis are known to be salt-sensitive in yeast and plants (Rausell *et al.*, 2003). For example, the expression of genes encoding the translation apparatus was lower when the transcriptome of Arabidopsis was compared with the halophyte salt cress (Taji *et al.*, 2004). If seagrass ribosomes are adapted to relatively high salinity, this may have an application for improvement of salt tolerance in crop species.

Sequence variations were identified in chloroplast-encoded 50S ribosomal protein L16. Nine amino acid mutations were shared by the three seagrass species despite belonging to two separate clades (Les *et al.*, 1997; Li and Zhou, 2009; Les and Tippery, 2013; Petersen *et al.*, 2016; Ross *et al.*, 2016). The possible convergence is highlighted by the absence of these mutations in representatives of sister genera for both clades. *Potamogeton perfoliatus* belongs to the tepaloid clade together with Zosteraceae, whereas *Najas flexilis*, *Elodea canadensis* and *Sagittaria lichuanensis* belong to the petaloid clade together with *H. ovalis* (Les *et al.*, 1997; Li and Zhou, 2009; Les and Tippery, 2013; Petersen *et al.*, 2016; Ross *et al.*, 2016). Protein sequences of L16 in these non-marine species have greater similarity with other monocots and dicots than with the seagrasses (Figs 2, 3) suggesting selection and convergent evolution to the marine habitat in seagrasses. Since *N. flexilis* and *P. perfoliatus* shared submergence characteristics with seagrasses, the mutations may be linked to salinity tolerance, rather than an ability to survive underwater. These results complement the seagrass clustering of OGCZ through OrthoMCL analysis and provided further molecular evidence of convergent evolution of seagrasses.

Differences between *H. ovalis* and the two Zosteraceae species were identified in genes encoding NDH, a major protein complex residing in the thylakoid membrane of chloroplasts that participates in cyclic electron flow pathways as an oxidoreductase (reviewed in Peltier *et al.*, 2016). As the NDH complex is only present in the Streptophyta lineage, which includes charophyte algae and land plants, acquisition of novel NDH genes likely occurred during terrestrial transition, and NDH is hypothesized to be one of the innovations enabling land plant evolution (Martín *et al.*, 2009; Ruhlman *et al.*, 2015). The absence of genes encoding NDH subunits and proteins required for complex formation in *H. ovalis* points to a total loss of the NDH complex in the *H. ovalis* thylakoid. Rare evidence of loss or pseudogenization of plastid NDH genes has been reported in independent lineages (Wolfe *et al.*, 1992; Haberhausen and Zetsche, 1994; Funk *et al.*, 2007; Braukmann *et al.*, 2009; Logacheva *et al.*, 2011), including several genera in the Hydrocharitaceae family (Iles *et al.*, 2013; Peredo *et al.*, 2013; Wilkin and Mayo, 2013; Ross *et al.*, 2016). The observed loss of NDH genes in *H. ovalis* is the first report of their dispensability among Alismatales.

Several reasons for NDH dispensability have been suggested (Stefanović and Olmstead, 2005; Iles *et al.*, 2013; Peredo *et al.*, 2013; Xu *et al.*, 2013). Ross *et al.* (2016) suggest that NDH loss enabled low N investment as an adaptation to nutrient deficiency in the submerged environment. This is plausible, as *H. ovalis* is adapted to grow in low nutrient sediments (Carruthers *et al.*, 2007), and examples of nitrogen sources affecting NDH expression have been reported in green algae (Peltier and Schmidt, 1991). Interestingly, two proteins related to nitrate uptake, nitrogen reductase 1 (NR1) and nitrate transporter (NRT3.1) are also lost in *H. ovalis* (Table S4). One limitation of seagrass nitrogen uptake studies is that the potential contribution of microbial communities is not accounted for, and may be compensating for the loss of NDH in *H. ovalis*, as demonstrated in myco-heterotrophic liverworts (Wickett *et al*., 2008*a*,b). Cyanobacteria on leaves have been shown to contribute to nitrogen uptake in *Posidonia* (Jeremy Bougoure, personal communication). However, further targeted studies are required to determine whether the loss of the NDH complex in *H. ovalis* is related to nitrogen uptake.

## Conclusion

Together, the conservation of gene loss and the sharing of seagrass-specific orthologues in these two independent lineages, despite the phylogenetic distance, has shed light on the genetics of marine adaptation in angiosperms of land plant ancestry. These results also present another example of habitat-driven parallel evolution in the plant kingdom.

## Supplementary data

Supplementary data are available at *JXB* online.

Fig. S1. Venn diagram showing the number of shared orthologous clusters among six species (Arabidopsis, *M. acuminata*, *O. sativa*, *S. polyrhiza*, and two Zosteraceae species).

Table S1. Species selected for multiple sequence alignment of orthologous proteins.

Table S2. Number of *H. ovalis* reads sequenced and remaining after filtering process.

Table S3. List of TAIR genes that were conserved in OGCsM and at least one species among *H. ovalis*, *Z. muelleri*, and *Z. marina*.

Table S4. List of TAIR genes that were conserved in OGCsM but absent in at least one species among *H. ovalis*, *Z. muelleri*, and *Z. marina*.

Table S5. Significantly enriched biological process GO terms in the genes that were lost in *H. ovalis*, but present in *Z. muelleri*, *Z. marina*, and five other plant species.

Table S6. List of orthologous groups that are conserved in *Z. muelleri* and *Z. marina* (OGCZ).

Table S7. List of OGCZ orthologous groups that are conserved in *H. ovalis* and the best corresponding TAIR ID hit; each protein ID is followed by InterProScan IDs of domains found or no domain found (NA).

Supplementary Tables

Supplementary Figure

## Abbreviations:

Mya: million years ago
NDH: NADH dehydrogenase-like
OGC: orthologous gene cluster
OGCsM: orthologous gene cluster with at least one gene originating from a monocot species
OGCZ: orthologous gene cluster unique to Zosteraceae
TAIR: The Arabidopsis Information Resource.
