# Supplementary material for: Genomic comparison of two independent seagrass lineages reveals habitat-driven convergent evolution
Source: J Exp Bot. 2018 Apr 18;69(15):3689–702. doi: 10.1093/jxb/ery147 (PMC6022596; doi:10.1093/jxb/ery147)
Supplement: Supplementary Figure [file ery147_suppl_supplementary_figure_s1.pdf]

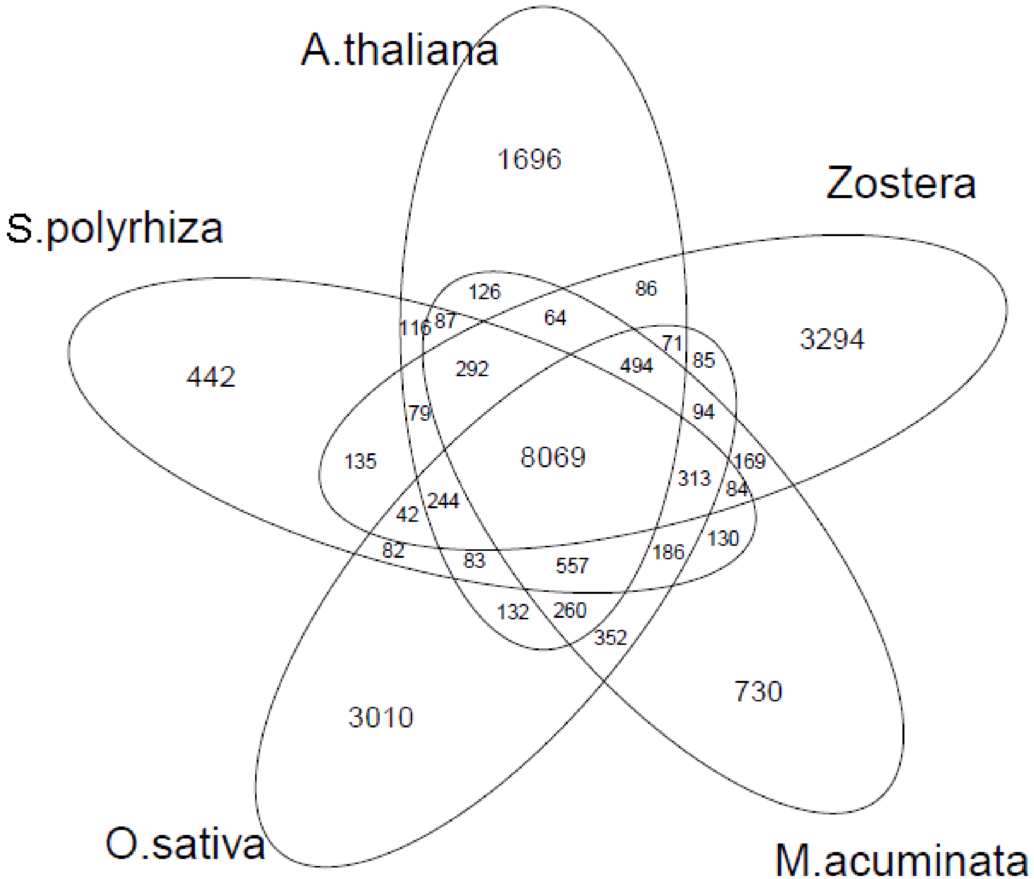

Figure S1. Venn diagram showing the number of shared orthologous clusters among six species (*A. thaliana*, *M. acuminata*, *O. sativa*, *S. polyrhiza* and two Zosteraceae species).
